# Purification and characterization of thermoactive serratiopeptidase from *Serratia marcescens* AD-W2

**DOI:** 10.1186/s13568-021-01215-7

**Published:** 2021-04-09

**Authors:** Devtulya Chander, Jasmine Kour Khosla, Diksha Koul, Md. Mehedi Hossain, Mohd Jamal Dar, Asha Chaubey

**Affiliations:** 1 Fermentation Technology Division, CSIR-Indian Institute of Integrative Medicine, Canal Road, Jammu, 180001 India; 2 Cancer Pharmacology Division, CSIR-Indian Institute of Integrative Medicine, Canal Road, Jammu, 180001 India; 3 Academy of Scientific and Innovative Research, CSIR-Human Resource Development Centre, Campus Ghaziabad, Ghaziabad, 201002 India

**Keywords:** *Serratia marcescens*, Metalloprotease, Serratiopeptidase, Serralysin, Homology modelling

## Abstract

Serratiopeptidase is a proteolytic enzyme extensively used as an anti-inflammatory and analgesic drug. Present work reports a thermoactive serratiopeptidase from *Serratia marcescens* AD-W2, a soil isolate from the North-Western Himalayan region of India. The extracellular metalloprotease has been purified by a simple two-step procedure resulting in a specific activity of 20,492 Units/mg protein with 5.28-fold purification. The molecular mass of the metalloprotease, as determined by SDS-PAGE was ~ 51 kDa. The purified serratiopeptidase presented optimum activity at pH 9.0, temperature 50 °C and stability in wide pH and temperature range. Critical temperature of 50 °C confirmed the thermoactivity of the purified serratiopeptidase. The kinetic studies of the purified serratiopeptidase revealed V_max_ and K_m_ of 57,256 Units/mL and 1.57 mg/mL, respectively, for casein. The purified serratiopeptidase from *S. marcescens* AD-W2 was found to be 100% identical to serralysin from *Serratia marcescens* ATCC 21074/E-15. The catalytic domain comprising of Zn coordinated with three histidine residues (His192, His196, His202), along with glutamate (Glu193) and tyrosine (Tyr232) residues, further confirmed that the purified protein is identical to serralysin.

**Supplementary Information:**

The online version contains supplementary material available at 10.1186/s13568-021-01215-7.

## Key points

- Thermoactive serratiopeptidase from *S. marcescens* AD-W2 was purified with simple two step purification method.
- Purified serratiopeptidase has specific activity of 20,492 Units/mg protein and critical temperature of 50 °C.
- Mass fingerprint and predicted catalytic site confirms that the purified protein is serralysin.

## Introduction

Serratiopeptidase (EC.3.4.24.40), also known as serrapeptase or serralysin, is an extracellular metalloprotease enzyme known for its pharmaceutical importance. It is widely used as an anti-inflammatory, analgesic, anti-oedemic, and anti-biofilm formulation agent (Ethiraj and Gopinath 2017). Serratiopeptidase has gained attention as a potent analgesic and anti-inflammatory drug with rapidly increasing market demands in the recent past (Olmstead 2017; Olmstead 2018; Srivastava et al. 2019). This drug also finds applications in chronic inflammatory diseases such as fibrocystic breast disease, sinusitis, carpal tunnel syndrome, bronchitis, arthritis, and atherosclerosis (Pakhale and Bhagwat 2016).

Serratiopeptidase was first isolated from Enterobacterium *Serratia* E-15, an isolate from the gut of *Bombyx mori* (Miyata et al. 1970). Serratiopeptidase is reportedly produced from *S. marcescens*, a Gram-negative small rod belonging to the family Enterobacteriaceae. Although *S. marcescens* is one of the most common serratiopeptidase producers (Romero et al. 2001; Nam et al. 2013; Ethiraj and Gopinath 2017), other bacteria have also been reported for serratiopeptidase production. These include other *Serratia* sp., *Bacillus licheniformis*, *Streptomyces hydrogenans* (Salarizadeh et al. 2014; Wagdarikar et al. 2015; Nageswara et al. 2019).

Keeping in view, the increasing demand of anti-inflammatory therapeutics in the global market, there is an immediate need to evolve efficient processes for serratiopeptidase production with efficient activity. In this context, our group has been engaged in isolation and characterization of serratiopeptidase producing microorganisms from NW Himalayan region and has recently reported *S. marcescens* isolated from mulberry phyllosphere for serratiopeptidase production (Koul et al. 2020). The present study relates to the isolation, characterization and purification of serratiopeptidase from *S. marcescens* AD-W2, a soil isolate from NW Himalayas. The purified serratiopeptidase has been investigated for its characteristics and structural properties vis-a-vis serralysin from *S. marcescens* ATCC 21074/E-15.

## Materials and methods

### Isolation and characterization of *S. marcescens* AD-W2

*Serratia marcescens* AD-W2, used in the present study was isolated from soil, collected from NW-Himalayas at an altitude of about 1000ft by serial dilution method (James and Natalie 2014), and was maintained on tryptone soya agar (TSA). The culture was identified by 16S rRNA gene sequencing and the sequence was submitted in NCBI GenBank. The phylogenetic tree was made using the minimum evolution method (Rzhetsky and Nei 1992), using MEGA X (Kumar et al. 2018). The culture has been deposited in Sir Col. R. N. Chopra Microbial Resource Centre, Jammu, India, with accession number MRCJ-997.

### Serratiopeptidase production from *S. marcescens* AD-W2

The isolated microorganism was subjected to growth in a 500 mL Erlenmeyer flask containing 100 mL tryptone soya broth in a rotary shaker at 200 RPM at 30 °C. In order to evaluate the production of enzyme, several production media were used for enzyme production followed by evaluation of protease activity up to 72 h. Based on the screening of various production media, soyabean meal and casein based medium (constituents in g/L: Soyabean meal—20.0, Casein—15.0, dihydrogen ammonium phosphate—7.5, soya oil—5.0, NaCl—0.5, KCl—0.1, MgSO_4_—0.1, ZnSO_4_—0.1, dextrose anhydrous-20.0, pH 7.5) was selected for protease production for further studies.

### Extracellular protease assay and protein estimation

The qualitative protease assay of the culture supernatant was performed on the milk agar plate (James and Natalie 2014), while the quantitative assay was performed according to Cupp-Enyard (2008) with slight modifications. One Unit of Protease (serratiopeptidase) activity was defined as micrograms of tyrosine released per minute on hydrolysis of casein under standard assay conditions (pH 8.0, 37 °C). Protein estimation was performed by the Bradford method (Bradford 1976).

### Purification of serratiopeptidase from *S. marcescens* AD-W2

The culture supernatant having the desired specific activity was partially purified by ammonium sulphate up to 30–80% saturation. The precipitates were dissolved in 0.05 M buffer (pH 8.0), dialyzed against the same buffer for enzyme activity or protein content evaluation, and further subjected to Ion exchange chromatography using the MonoQ5/50 GL column (GE make) installed with Bio-Rad DuoFlow Chromatography system. 40 mg of partially purified protein was loaded onto the column, and elution was performed using NaCl gradient in phosphate buffer (pH 6.0). 0.5 mL fractions were collected and evaluated for protease (serratiopeptidase) activity.

### Sodium dodecylsulphate-polyacrylamide gel electrophoresis (SDS-PAGE) and zymography

The crude, partially purified and purified enzymes were subjected to 10% SDS-PAGE according to Laemmli discontinuous system (Laemmli 1970). Zymography was performed as per protocol given by Lantz and Ciborowski (1994) with few modifications.

### Characterization of serratiopeptidase from *S. marcescens* AD-W2

In order to evaluate the optimum activity, the assay was carried out, keeping all the assay conditions the same, except the variables (pH, temperature, substrate concentration, or inhibitors). The relative activities were calculated, considering the initial activity as 100%. The effect of temperature on serratiopeptidase activity was studied at 30–60 °C, while the effect of pH on the serratiopeptidase activity was evaluated at varied pH range, i.e., pH 6.0–7.0 (phosphate buffer), 8.0–9.0 (Tris–HCl buffer), pH 10.0 (Glycine NaOH), and pH 11.0–12.0 (Sodium bicarbonate buffer). The effect of inhibitors was studied with 2 mM inhibitors (Olajuyigbe and Falade 2014). The kinetic studies of purified serratiopeptidase were performed by activity assay with varying concentrations of casein under standard assay conditions to determine V_max_. K_m_ values were determined by the double reciprocal Lineweaver–Burk plot. The critical temperature of the purified serratiopeptidase and activation energies below and above critical temperatures were determined by the Arrhenius plot generated from the temperature-activity dataset.

### Identification of purified serratiopeptidase

The purified protein band from the SDS-PAGE gel was trypsin digested by the procedure discribed in Sigma Proteoprep kit, while reduction and alkylation steps were omitted for rapid processing (Shevchenko et al. 2006). The mass fingerprints of the peptides were obtained on Bruker UltrafleXtreme MALDI TOF/TOF using multiple laser shots. 2 µL of the tryptic digest was mixed with α-cyano-4-hydroxycinnamic acid (10 mg/mL CHCA in 50% acetonitrile and 0.1% TFA) in a 1:1 ratio, transferred on the target MALDI plate and was allowed to dry at room temperature. Bovine serum albumin was used as a positive control. The mass spectra were searched in the Swiss-prot database along with the contaminant database on the MASCOT server for identification of the protein (Perkins et al. 1999). The sequence obtained using the MASCOT software after MALDI-TOF analysis was subjected to BLAST in the UNIPROT database. The top six results were used for multiple sequence alignment in SEAVIEW software (Galtier et al. 1996). The three-dimensional structure of serratiopeptidase was built using the SWISS-MODEL server (Schwede et al. 2003) using serralysin protein from *Serratia* sp. (PDB entry 1srp) as the template.

## Results

### Isolation and characterization of *S. marcescens* AD-W2

**Fig. 1:**
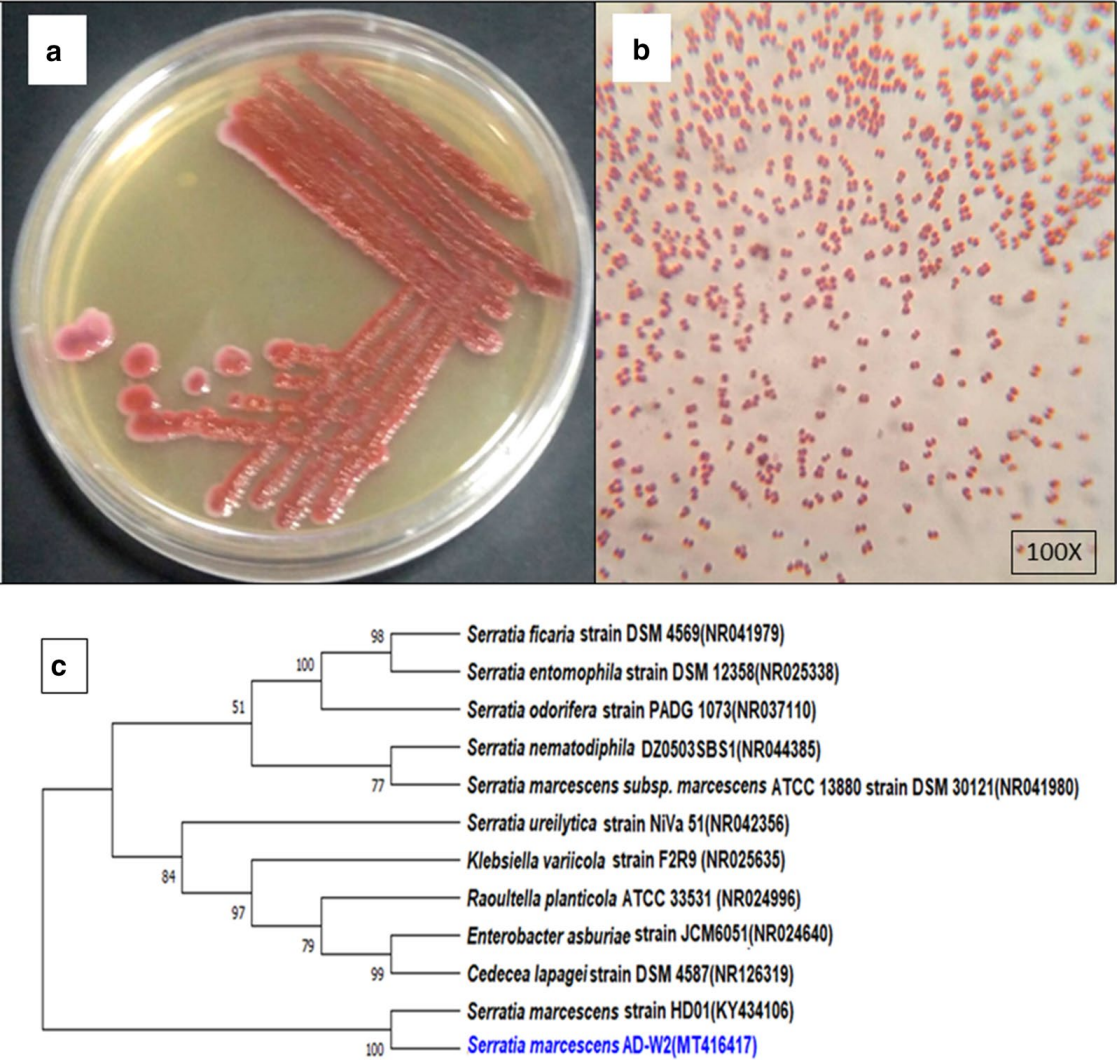
**a** Morphology of pure isolate AD-W2 on Nutrient Agar plate. **b** Microscopic image of pure isolate AD-W2 after Gram staining. **c** Phylogenetic tree of AD-W2 with closely related 16S rRNA gene sequences of Type strains

The isolated strain AD-W2 presented the characteristic morphological features of *Serratia* sp. (Zhang et al. 2009) like red pigmentation, short Gram-negative rod shape are shown in Fig. 1a, b. The 16S rRNA gene sequence revealed that the isolate has a 99.85% similarity with both *S. nematodiphila* and *S. marcescens* type strain. The negetive results for lactose utilization, xylose utilization, and arginine dihyrolase, confirmed that the isolate is *S. marcescens* and not *S. nematodiphila.* The sequence was submitted to the NCBI GenBank with Accession number MT416417. As per the phylogenetic tree made using the Minimum evolution method in Fig. 1c, the strain had the highest similarity with *S. marcescens*. The culture has been deposited in Sir R. N. Chopra, Microbial Resource Centre Jammu, India with Accession number MRCJ-997.

### Purification of serratiopeptidase from *S. marcescens* AD-W2

**Fig. 2:**
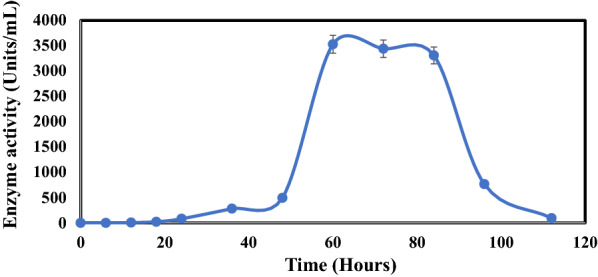
Time course profile of *S. marcescens* AD-W2 for serratiopeptidase production

**Fig. 3:**
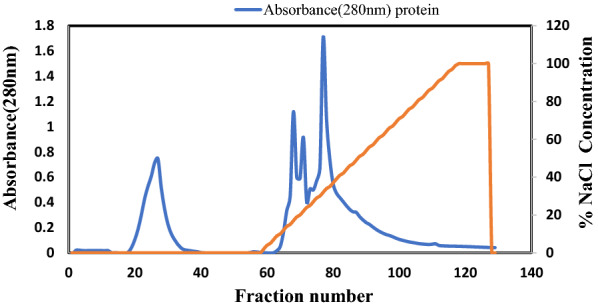
The protein elution profile during Ion exchange chromatography using MonoQ 5/50 GL column

Serratiopeptidase production profile of *S. marcescens* AD-W2 in the production medium has been demonstrated in Fig. 2. The maximum extracellular protease activity of 3528 Units/mL was obtained in 60 h. Partial purification by ammonium sulphate precipitation at 30–80% saturation led to the specific activity of 7659 Units/mg protein. The compilation of purification of serratiopeptidase from *S. marcescens* AD-W2 along with specific activities and purification folds, has been shown in Table 1. The purification of around two fold was achieved in ammonium sulphate precipitation. However, Romero et al. (2001) achieved 3.6 fold purification after 50–70% ammonium sulphate saturation. Further purification by MonoQ ion-exchange chromatography (Fig. 3) resulted in the specific activity of 20,492 (Units/mg protein) with a purification fold of 5.28.

**Table 1:** The parameters of purification of serratiopeptidase from culture supernatant up to ion-exchange chromatography

Sample	Total protease unit	Total protein content (mg)	Specific activity (units of enzyme activity/mg protein)	Purification fold
Culture supernatant	613,851.8	158.3	3877.466	1
Ammonium sulphate (40–80%)	49,156.85	6.42	7658.901	1.97
Ion exchange chromatography (MonoQ column)	39,533.85	1.93	20,492.59	5.28

### SDS-PAGE and zymography of serratiopeptidase from *S. marcescens* AD-W2

**Fig.4:**
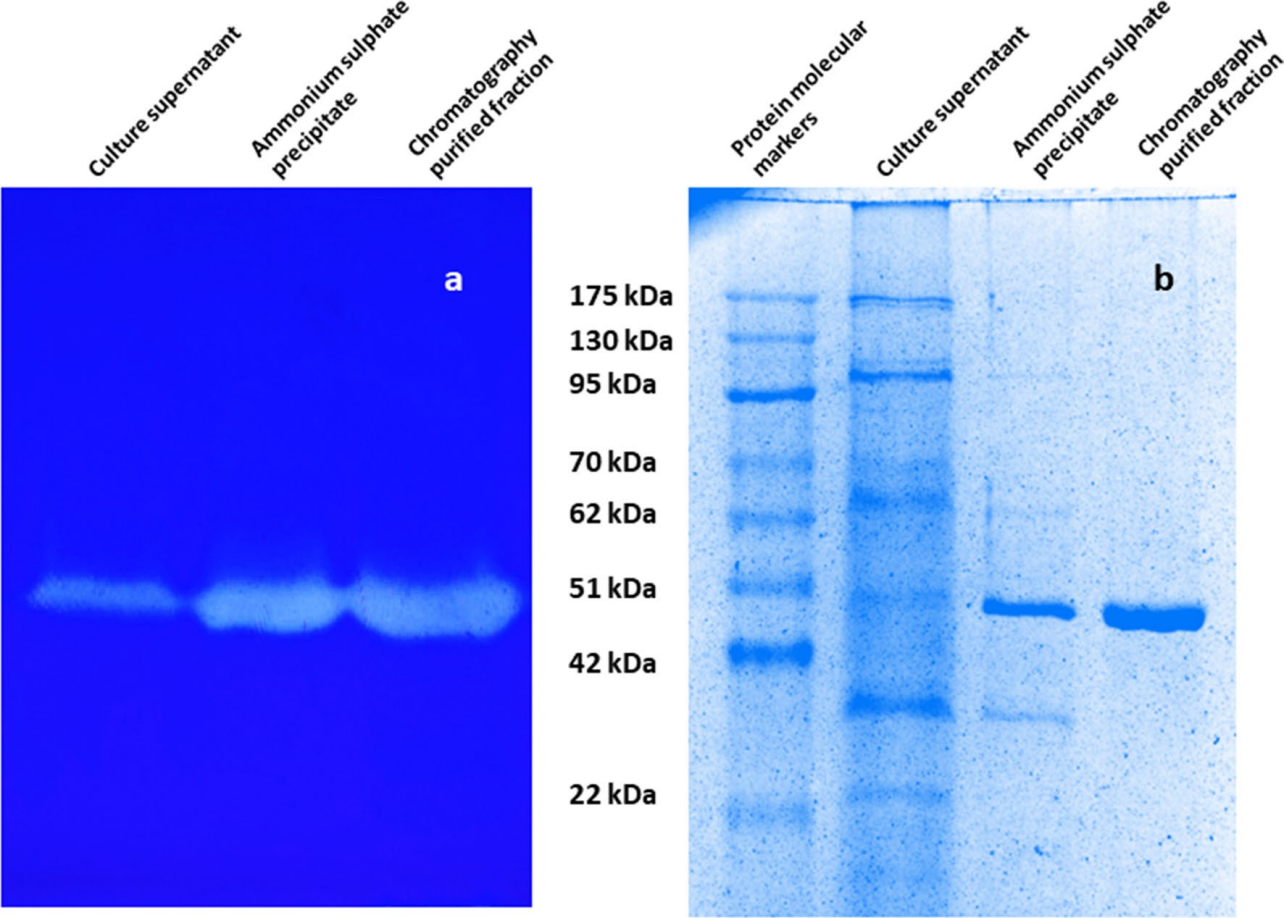
**a** Casein Zymogram and **b** SDS-PAGE of serratiopeptidase from *S. marcescens* AD-W2

SDS-PAGE of crude extracellular protease, partially purified and purified serratiopeptidase, has been demonstrated in Fig. 4. Zymogram of the crude culture supernatant, partially purified and purified serratiopeptidase indicates that the purified protein is indeed protease with clear zones appeared due to hydrolysis of the substrate (casein). The molecular weight of purified serratiopeptidase molecule was predicted to be ~ 51 kDa, which is in the range of other serratiopeptidase reported from various sources (Devi et al. 2013; Nam et al. 2013; Gupte and Luthra 2017; Ethiraj and Gopinath 2017).

### Characterization of purified serratiopeptidase from *S. marcescens* AD-W2

**Fig. 5:**
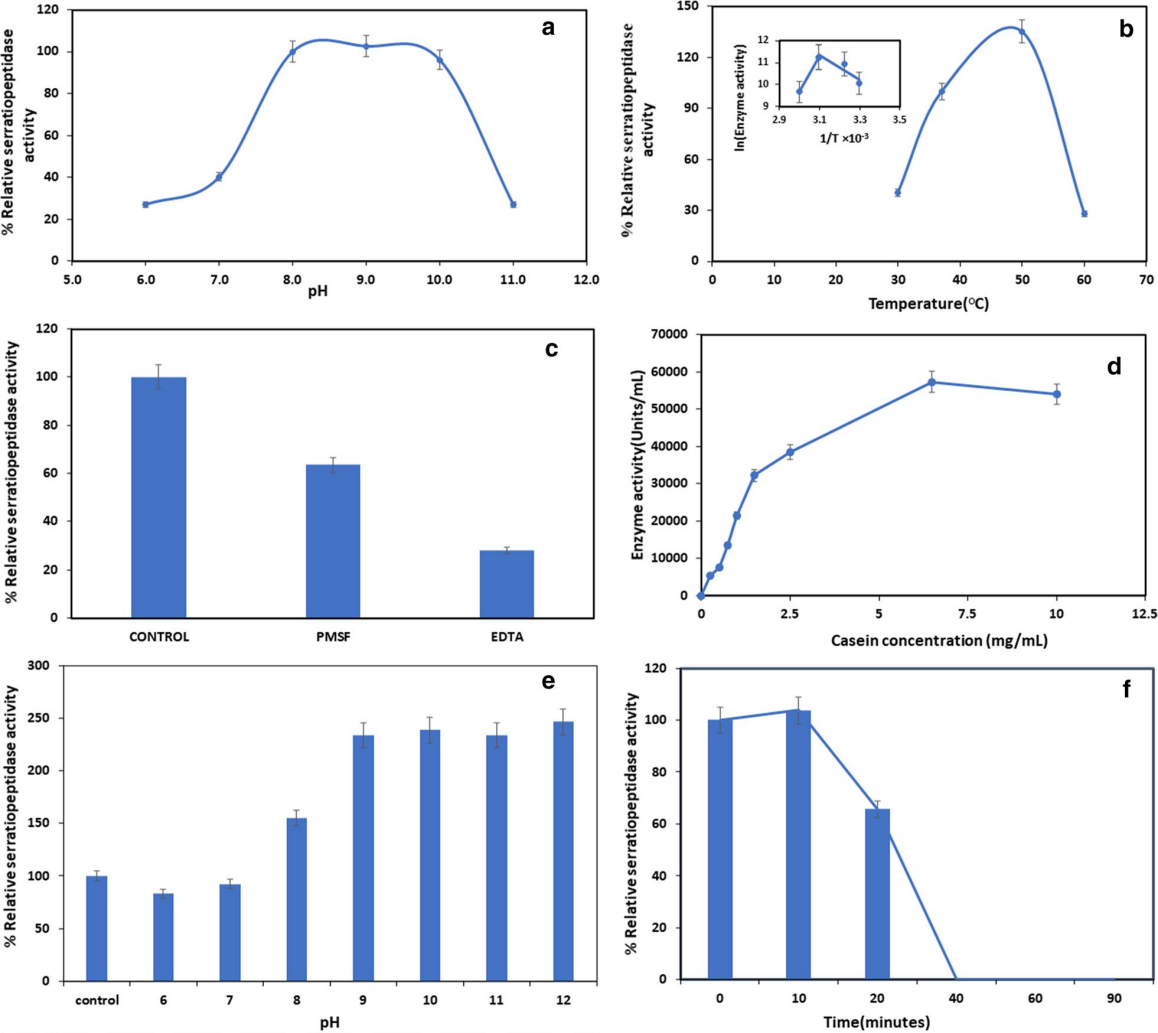
Effect of various reaction conditions and stability of serratiopeptidase from *S. marcescens* AD-W2: **a** pH; **b** temperature (Arrhenius plot shown in inset); **c** inhibitors **d** substrate concentration; **e** pH stability; **f** temperature stability at 50 °C

The characteristics of purified serratiopeptidase from *S. marcescens* AD-W2 have been demonstrated in Fig. 5. The optimum pH (Fig. 5a) and temperature (Fig. 5b) of serratiopeptidase from *S. marcescens* AD-W2 were observed to be pH 9.0 and 50 °C, respectively. Critical temperature, as determined by the Arrhenius plot (Fig. 5b inset), was 50 °C, above which the enzyme gets inactivated. The activation energy for the reaction to proceed below critical temperature, i.e., 50 °C, was − 45 kJ/mol, while above critical temperature, it was calculated as 141 kJ/mol. The inhibition studies indicate that EDTA inhibits 71.2% activity (Fig. 5c). However, inhibition by PMSF was not significant. As shown in Fig. 5d, The activity increased with substrate concentration until 6.5 mg/mL, thereafter it did not increase. V_max_ and K_m_ values of purified serratiopeptidase from *S. marcescens* AD-W2 are 57,256 Units/mL and 1.57 mg/mL, respectively, for casein. The stability studies indicate that the purified serratiopeptidase is stable under a wide temperature range up to 50 °C and pH range with higher conformational stability at higher pH (Fig. 5e). The stability profile also indicates that the purified serratiopeptidase retains 65% activity after 20 min incubation at 50 °C and 15% at 55 °C activity after 10 min incubation (Fig. 5f).

### Identification of purified serratiopeptidase

The protein mass fingerprint (Additional file 1: Fig. S1) was searched in the MASCOT database and the search report revealed that the purified protein from *S. marcescens* AD-W2 was similar to serralysin (UNIPROT Accession number P07268) with 65% sequence coverage and a negligible chance of being false. The protein scores and sequence coverage of serratiopeptidase from *S. marcescens* AD-W2 with the reported serralysin are available in the (Additional file 1: Figs. S2 and S3).

**Fig. 6:**
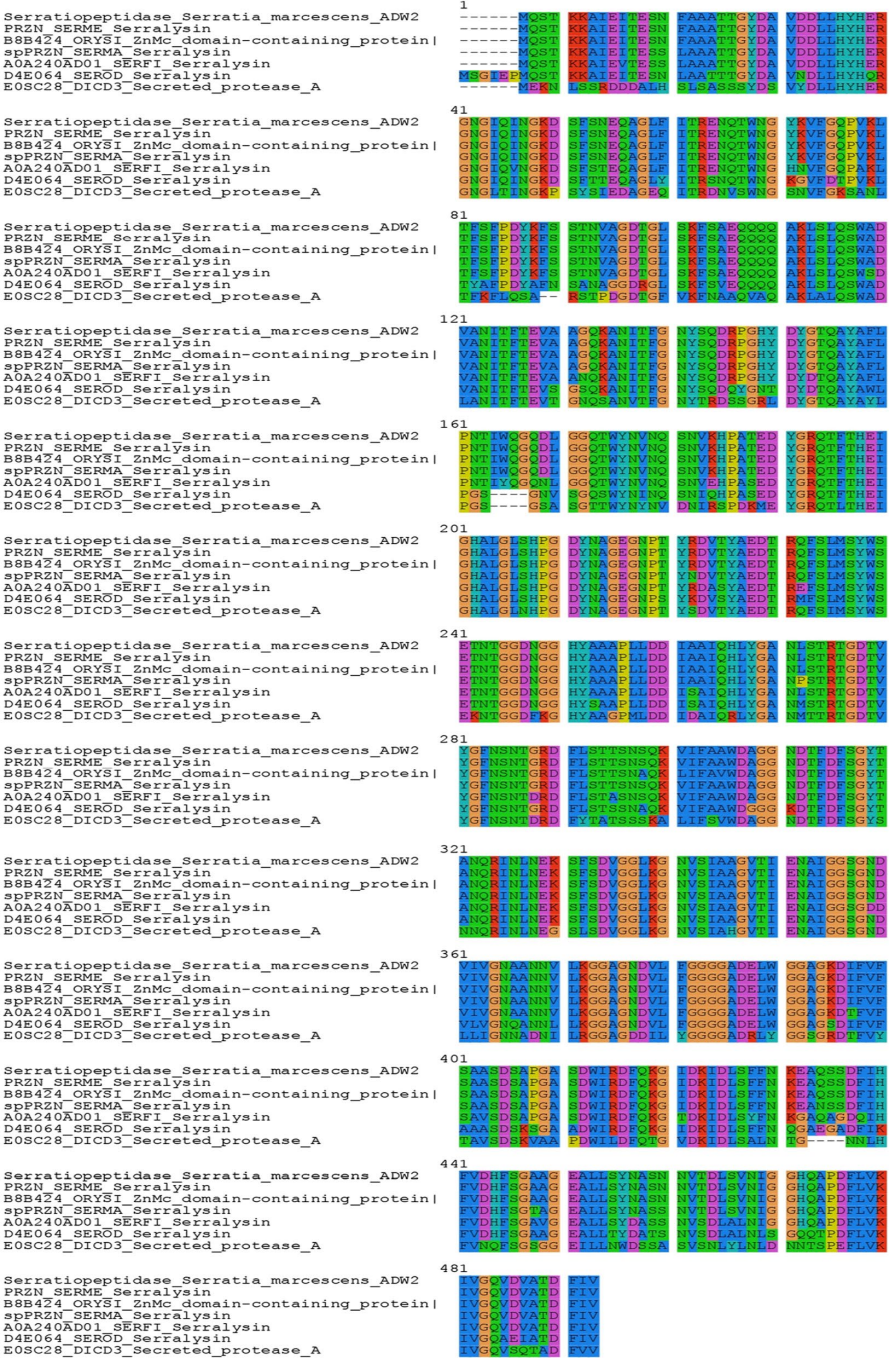
Multiple amino-acid sequence alignment of the serratiopeptidase from *S. marcescens* AD-W2, PRZN_SERME serralysin from *S. marcescens* (E-15) with 100% sequence identity, PRZN_SERMA Serralysin from *S. marcescens* with 98.6% sequence identity, 0A240AD01_SERFI Serralysin from *S. ficaria* with 92.6% sequence identity, D4E064_SEROD Serralysin from *S. odorifera* DSM 4582 with 82.8% sequence identity and E0SC28_DICD3 Secreted protease A from *Dickeya dadantii* with 64.8% sequence identity. The protein sequence from serratiopeptidase was aligned to see the similarities, differences, and conservation of various amino acid residues using multiple sequence alignment. The alignment was carried out by using Clustal W platform in Seaview software

**Fig. 7:**
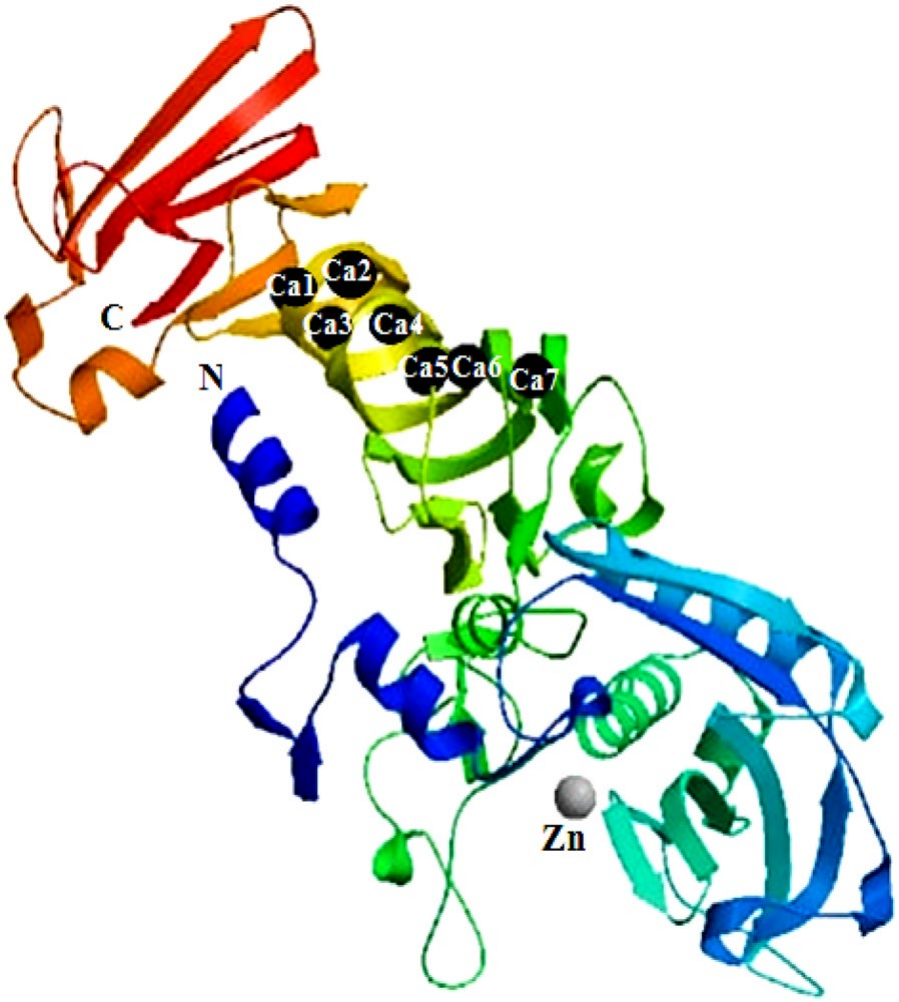
Cartoon view of the overall three-dimensional structure of Serratiopeptidase, a ribbon diagram showing the positions of the secondary structure elements was built using online SWISS-MODEL software (https://swissmodel.expasy.org/) and colored according to secondary-structure element. β-Strands are depicted as arrows, the zinc ion is shown by a grey ball, and calcium ions by black balls

**Fig. 8:**
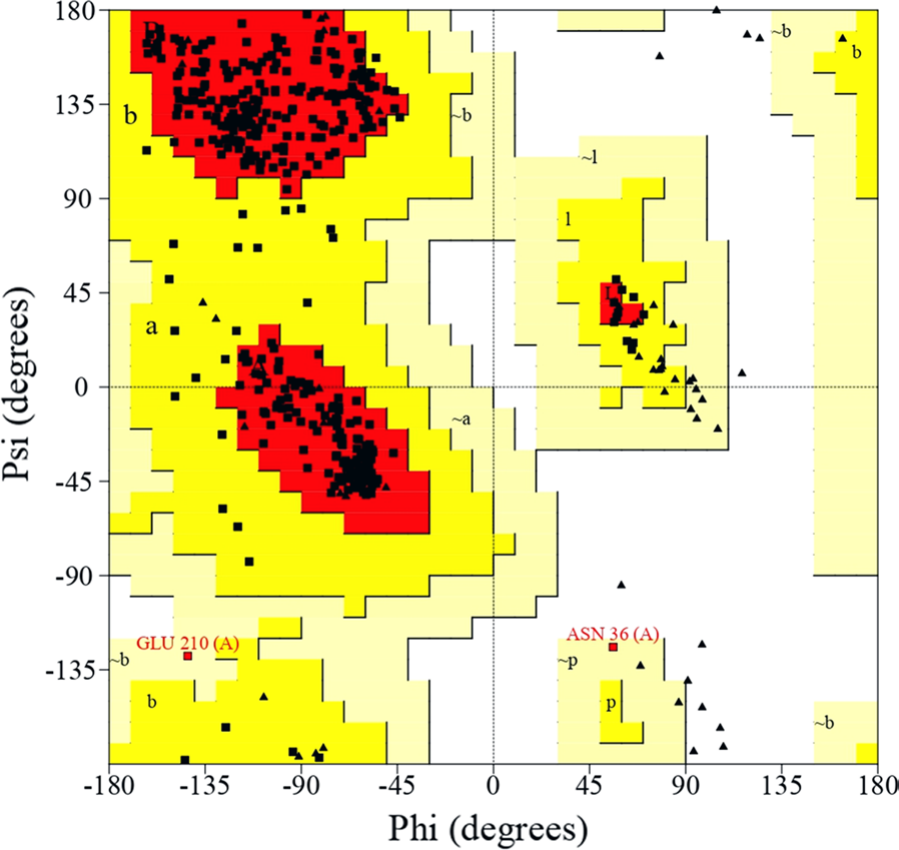
Ramachandran plot of the chimeric protein. The Ramachandran plot analysis (PROCHECK server); Laskowski et al. (1993), revealed that the modelled structure projected 91.0% of amino acid residues in most favoured regions of the Ramachandran plot, 8.5% are in additional allowed regions, 0.5% residues in generously allowed regions and 0.0% residues in disallowed regions

**Fig. 9:**
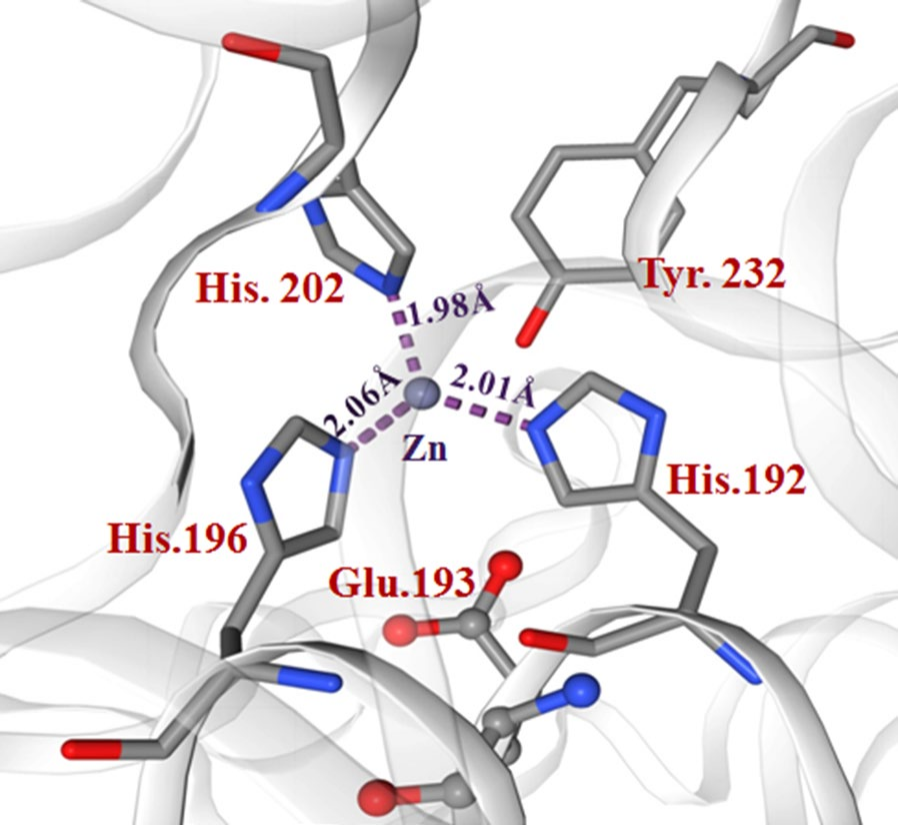
Stereoview of the zinc-binding site in the Serratiopeptidase. The zinc ion is shown as a purple ball. The active site of serratiopeptidase from *S. marcescens* AD-W2 shows the zinc ion coordinated as Zn.1, His.192, His.196, His.202, Glu.193, and Tyr.232 is forming a distorted trigonal bipyramidal catalytic active center

The multiple sequence alignment of serralysin from different sources with the sequence of purified protein from *S. marcescens* AD-W2 has been shown in Fig. 6. The three-dimensional homology model of serratiopeptidase from *S. marcescens* AD-W2, as shown in Additional file 1: Fig. S1, was generated using online SWISS-MODEL software (http://swissmodel.expasy.org/) (Schwede et al. 2003) (Fig. 7). The template used by the modeller for building model was the crystal structure of serralysin protein from *Serratia* sp. (PDB entry 1srp), the first serralysin reported and characterized (Hamada et al. 1996). The biocatalytic qualities of the predicted models were confirmed by the PROCHECK server.  Figure 8 demonstrates the Ramachandran plot analysis of the model protein. The stereo view of the catalytic domain (Fig. 9), from the depicted catalytic domain of serratiopeptidase from *S. marcescens* AD-W2 comprises of Zinc coordinated with three main residues, i.e. His192, His196, His202 along with Glu193 and Tyr232.

## Discussion

Serratiopeptidase is an industrially important enzyme being used in the pharmaceutical industry as therapeutics (Ethiraj and Gopinath 2017). *S. marcescens* is known as the largest producer of serratiopeptidase. However, other bacteria are also known for Serratiopeptidase production (Salarizadeh et al. 2014; Wagdarikar et al. 2015; Nageswara et al. 2019). During the present study, *S. marcescens* AD-W2 has been demonstrated as a source of thermoactive serratiopeptidase. Although, the strain has a similarity with *S. marcescens* as well as *S. nematodiphila*, the biochemical tests like lactose utilization, xylose utilization, and arginine dihyrolase test revealed its close relation with *S. marcescens* (Zhang et al. 2009). The phylogenetic tree generated by the minimum evolution method also confirmed our observation to conclude the identification of the isolate as *S. marcescens*.

The serratiopeptidase from *S. marcescens* AD-W2 was purified by a simple two-step procedure presenting the specific activity of 20,492 Units/mg protein with an overall 5.28-fold purification. The molecular weight of purified serratiopeptidase molecule was found to be ~ 51 kDa, which is in the range of other serratiopeptidase reported from various sources (Devi et al. 2013; Nam et al. 2013; Gupte and Luthra 2017; Ethiraj and Gopinath 2017). Further, the zymogram confirmed that the purified protein was indeed the protease enzyme.

The characterization of the purified enzyme was carried out for optimum pH & temperature and the results are in agreement with previously demonstrated optimum pH from 8.5 to 10.0 (Miyata et al. 1970; Salamone and Wodzinski 1997; Nam et al. 2013; Salarizadeh et al. 2014). However, the optimum temperature was found to be higher than the previously reported temperature optima (Salamone and Wodzinski 1997; Nam et al. 2013; Salarizadeh et al. 2014). This is also higher than 40 °C as reported by Miyata et al. (1970) from *Serratia marcescens* E-15.

An attempt was made to determine the critical temperature of serratiopeptidase from *S. marcescens* AD-W2, which was calculated by the Arrhenius equation as 50 °C, above which the enzyme gets inactivated. To the best of our knowledge, this was the first attempt to determine the critical temperature of serratiopeptidase. This could be because of the positive activation energy for the reaction to proceed below the critical temperature, i.e., 50 °C. The inhibition studies indicate significant inhibition by EDTA and insignificant inhibition by PMSF, which confirms that the enzyme is a metalloprotease. The results from enzyme kinetics studies corroborate with the previously reported serralysin-like metalloprotease (Nageswara et al. 2019). The stability studies indicate that the purified serratiopeptidase is stable under wide temperature and pH range with higher conformational stability at higher pH. The stability profile has been observed to be better than previous reports showing 15% activity after 20 min of incubation at 50 °C (Nam et al. 2013).

Homology modelling of purified protein was performed using the template from the crystal structure of serralysin protein from *Serratia* sp. PDB entry 1srp (Hamada et al. 1996). Ramachandran plot indicates that there were 91.0% residues in the most favourable region, 8.5% residues in the additional allowed region, and 0.5% in the generously allowed region. There were no residues in the disallowed region and, the predicted overall three-dimensional structure of serratiopeptidase from *S. marcescens* AD-W2 shares an identical fold (100% identity) with its homolog, serralysin from *Serratia* sp. E-15. The 3D structure of serratiopeptidase from *S. marcescens* AD-W2 is a monomer, identical to the template molecule comprising of two domains and an extended N-terminal tail (Hamada et al. 1996). The serratiopeptidase from *S. marcescens* AD-W2 was predicted to be an elongated molecule with ligands 7 × CA (Calcium Ion) and 1 × ZN (Zinc Ion). It consists of an extended N-terminal tail linked with the C-terminus and two main domains i.e. N-terminal proteolytic domain and a catalytic domain with a Zinc ion. The stereoview (Fig. 9) of the depicted catalytic domain of serratiopeptidase from *S. marcescens* AD-W2 comprises of Zinc coordinated with three main residues, i.e. His192, His196, His202 along with Glu193 and Tyr232 residues. The predicted active site also corroborates with that of the crystal structure of serralysin (Wu et al. 2016; Nageswara et al. 2019).

In conclusion, serratiopeptidase from *S. marcescens* AD-W2 was successfully purified with a specific activity of 20,492 units/mg. The thermoactive serratiopeptidase was found to have stability in wide pH and temperature range with K_m_ of 1.57 mg/mL and V_max_ of 57,256 Units/mL, respectively. The predicted active site in the catalytic domain re-confirmed that the purified protein from *S. marcescens* AD-W2 is a serratiopeptidase. Further, the process for the production of serratiopeptidase may be developed either using the wild-type strain or by developing the recombinant strain.

## Supplementary Information

**Additional file 1. Fig. S1:** MALDI-MS/MS mass fingerprint of purified serratiopeptidase from Serratia marcescens AD-W2.** Fig. S2:** Protein scores obtained from MASCOT server with MALDI-MS/MS mass fingerprint.** Fig. S3:** Protein sequence of the purified serratiopeptidase from Serratia marcescens AD-W2. The red colour region shows sequence coverage (65%) in peptide mass fingerprint obtained through peptide mass fingerprint searches in the Mascot server.** Fig. S4:** gene sequence of serralysin was translated into protein and then aligned with protein sequences having highest similarity in UNIPROT database, we could see that the Zinc binding domain is highly conserved in nature. Even in proteins predicted from the plant genome oryza sativa there is very high similarity with that from microbial sources. This might be an indication of close relation of Serratia marcescens in plants not only limited to mulberry (Koul et al. 2020) and Cucurbita pepa (Selvakumar et al. 2008) and also much greater role of serralysin as natural insecticidal in plants (Kaviyarasi and Suryanarayan 2016).

## Data Availability

The datasets generated during and/or analysed during the current study are available from the corresponding author on reasonable request.
