# Correction to: Purification and characterization of thermoactive serratiopeptidase from *Serratia marcescens* AD-W2

**DOI:** 10.1186/s13568-021-01223-7

**Published:** 2021-05-04

**Authors:** Devtulya Chander, Jasmine Kour Khosla, Diksha Koul, Md. Mehedi Hossain, Mohd Jamal Dar, Asha Chaubey

**Affiliations:** 1 Fermentation Technology Division, CSIR-Indian Institute of Integrative Medicine, Canal Road, Jammu, 180001 India; 2 Cancer Pharmacology Division, CSIR-Indian Institute of Integrative Medicine, Canal Road, Jammu, 180001 India; 3 Academy of Scientific and Innovative Research, CSIR- Human Resource Development Centre, Campus, Ghaziabad, 201002 India

## Correction to: AMB Expr (2021) 11:53https://doi.org/10.1186/s13568-021-01215-7

Following publication of the original article (Chander et al. 2021), the authors identified an error in the co-author family name. The correct co-author name should be “Jasmine Kour Khosla” instead of “Jasmine Kour Khousla”. This has been corrected with this erratum.

The original article (Chander et al. 2021) has been updated.

## References

[CR1] Chander D, Khosla JK, Koul D, Hossain MM, Dar MJ, Chaubey A (2021). Purification and characterization of thermoactive serratiopeptidase from *Serratia marcescens* AD-W2. AMB Express.

