# Supplementary material for: Purification and characterization of thermoactive serratiopeptidase from Serratia marcescens AD-W2
Source: AMB Express. 2021 Apr 9;11:53. doi: 10.1186/s13568-021-01215-7 (PMC8035408; doi:10.1186/s13568-021-01215-7)
Supplement: Supplementary file 1 — Additional file 1. Fig. S1: MALDI-MS/MS mass fingerprint of purified serratiopeptidase from Serratia marcescens AD-W2. Fig. S2: Protein scores obtained from MASCOT server with MALDI-MS/MS mass fingerprint. Fig. S3: Protein sequence of the purified serratiopeptidase from Serratia marcescens AD-W2. The red colour region shows sequence coverage (65%) in peptide mass fingerprint obtained through peptide mass fingerprint searches in the Mascot server. Fig. S4: gene sequence of serralysin was translated into protein and then aligned with protein sequences having highest similarity in UNIPROT database, we could see that the Zinc binding domain is highly conserved in nature. Even in proteins predicted from the plant genome oryza sativa there is very high similarity with that from microbial sources. This might be an indication of close relation of Serratia marcescens in plants not only limited to mulberry (Koul et al. 2020) and Cucurbita pepa (Selvakumar et al. 2008) and also much greater role of serralysin as natural insecticidal in plants (Kaviyarasi and Suryanarayan 2016). [file 13568_2021_1215_MOESM1_ESM.docx]

**Additional File**

**Purification and characterization of thermoactive Serratiopeptidase from *Serratia marcescens* AD-W2**

**Devtulya Chander^1,3^, Jasmine Kour Khosla^1^, Diksha Koul^1,3^, Md. Mehedi Hossain^2,3^, Mohd Jamal Dar^2,3^ and Asha Chaubey^1,3*^**

^1^Fermentation Technology Division, CSIR-Indian Institute of Integrative Medicine,

Canal Road, Jammu-180001 (India)

^2^Cancer Pharmacology Division, CSIR-Indian Institute of Integrative Medicine,

Canal Road, Jammu-180001 (India)

^3^Academy of Scientific and Innovative Research, CSIR- Human Resource Development Centre, Campus Ghaziabad-201002 (India)

**Purification and identification of serratiopeptidase from *Serratia marcescens* AD-W2**

The culture supernatant having the desired specific activity was partially purified by ammonium sulphate upto 30-80% saturation. The precipitates were dissolved in 0.05M buffer (pH 8.0), dialyzed against the same buffer for enzyme activity or protein content evaluation, and further subjected to Ion exchange chromatography using the MonoQ5/50 GL column (GE make) installed with Bio-Rad DuoFlow Chromatography system. 40mg of partially purified protein was loaded onto the column, and elution was performed using NaCl gradient in phosphate buffer (pH 6.0). 0.5mL fractions were collected and evaluated for protease (serratiopeptidase) activity. The crude, partially purified, and purified enzymes were subjected to 10% SDS-PAGE according to Laemmli discontinuous system (Laemmli 1970). The purified protein band from the SDS-PAGE gel was trypsin digested by the Sigma Proteoprep kit, while reduction and alkylation steps were omitted for rapid processing (Shevchenko et al. 2006). The mass fingerprints of the peptides were obtained on Bruker Ultraflextreme MALDI TOF/TOF using multiple laser shots.

The band of serratiopeptidase from SDS-PAGE was excised and digested using the protocol of sigma proteoprep kit with some modification, and the MALDI analysis for identification of protein was performed on Bruker Ultraflextreme MALDI TOF/TOF and Peptide mass fingerprint was searched on MASCOT as shown in Fig. S1.

**
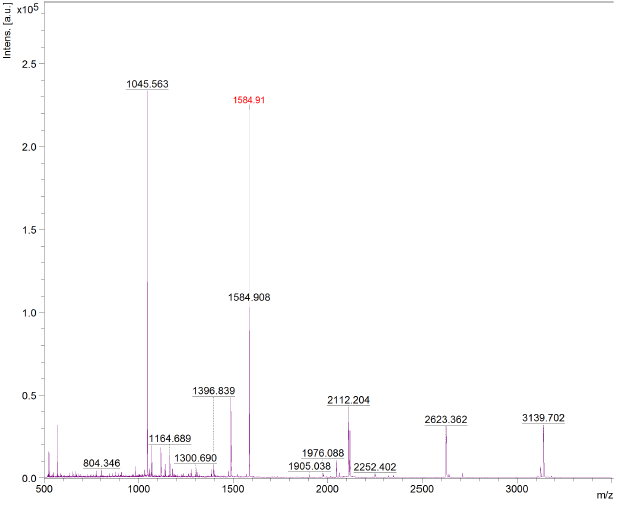
**

**Fig. S1** MALDI-MS/MS mass fingerprint of purified serratiopeptidase from *Serratia marcescens* AD-W2

The peptide mass fingerprint obtained (Fig. S1) thus was searched in the MASCOT server (Perkins et al. 1999), and the results are shown in Fig. S2.


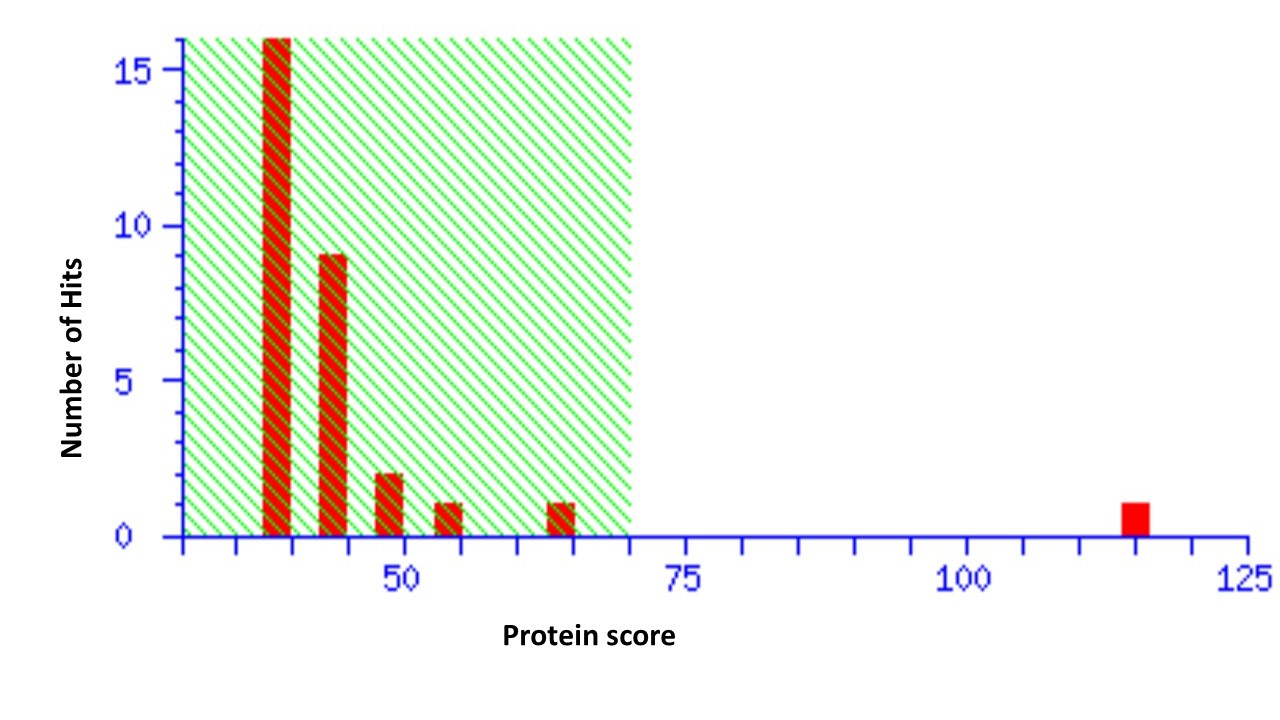


**Fig. S2** Protein scores obtained from MASCOT server with MALDI-MS/MS mass fingerprint

The protein scores of serratiopeptidase from S. *marcescens* AD-W2 with reported serralysin have been demonstrated in Fig.S2. The protein score was 115 depicting a 7.9e-5% chance of being false, which is negligible.


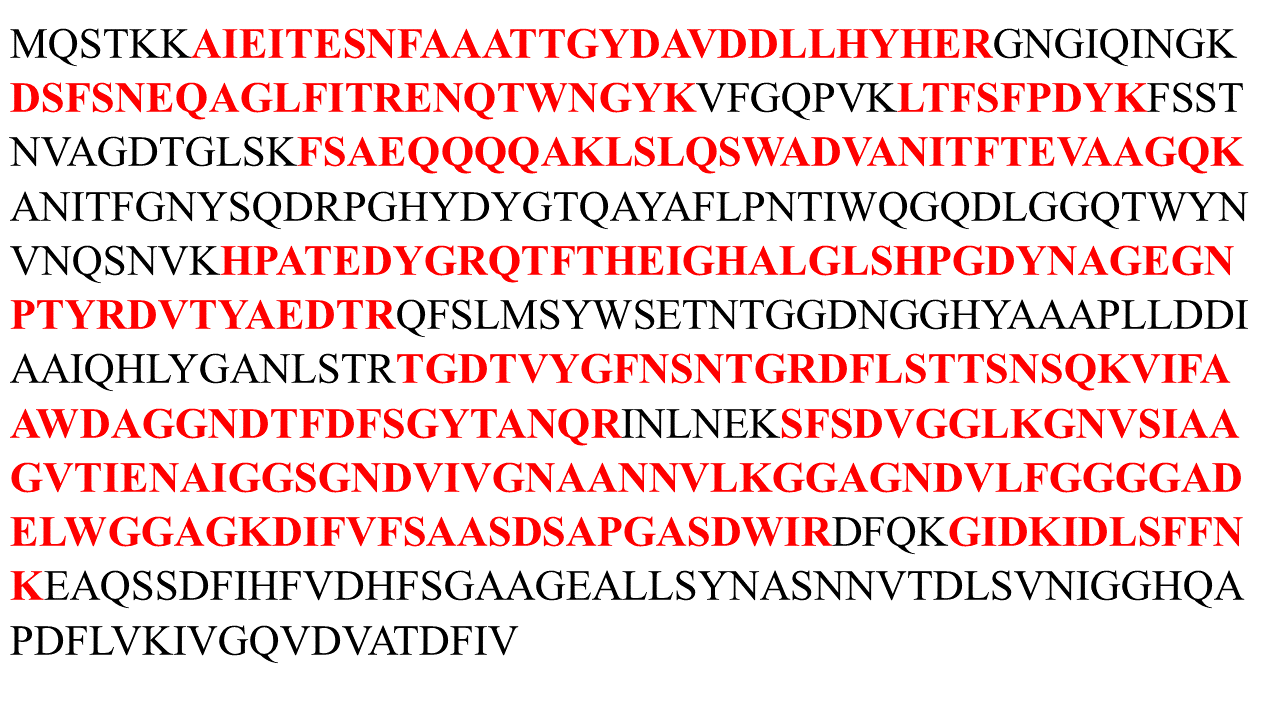


**Fig. S3** Protein sequence of the purified serratiopeptidase from *Serratia marcescens* AD-W2. The red colour region shows sequence coverage (65%) in peptide mass fingerprint obtained through peptide mass fingerprint searches in the Mascot server

**Table S1** The sequence of primers used as forward and reverse

| Primers | Sequence |
| --- | --- |
| Forward | TAT AAT ACT CAT ATG GCC GCG ACA ACCX |
| Reverse | ATG TAC CTC GAG TTA CAC GAT AAA GTCC |

The sequence of primers was taken from (Srivastava et al. 2019), and the reaction composition for 400µL of reaction was: Taq=2µL,10X PCR buffer( with MgCl2) =40 µL,dNTP mix=32 µL, template=16 µL, water= 302 µL, primer forward= 4 µL, primer Reverse =4 µL and stepwise PCR program was:-

1. 95°C- 5min
2. 94°C- 45 seconds
3. 59°C-1 minute
4. 72 °C- 1 minute 30 seconds
5. Repeat 2 to 4 34 times
6. 72°C-7 minute
7. 4°C-infinity

The PCR product was sequenced using the big dye terminator V3.1 based chemistry with given PCR condition; the terminated products were purified using the salt precipitation and then analyzed in applied biosystems 3730 XL genetic analyzer. The AB1 file thus generated was used to generate a consensus sequence in Tracy package (Rausch et al. 2020). The gene sequence was thus translated into protein, the blast was performed in Uniprot, and the multiple alignment was performed in the seaview software package.


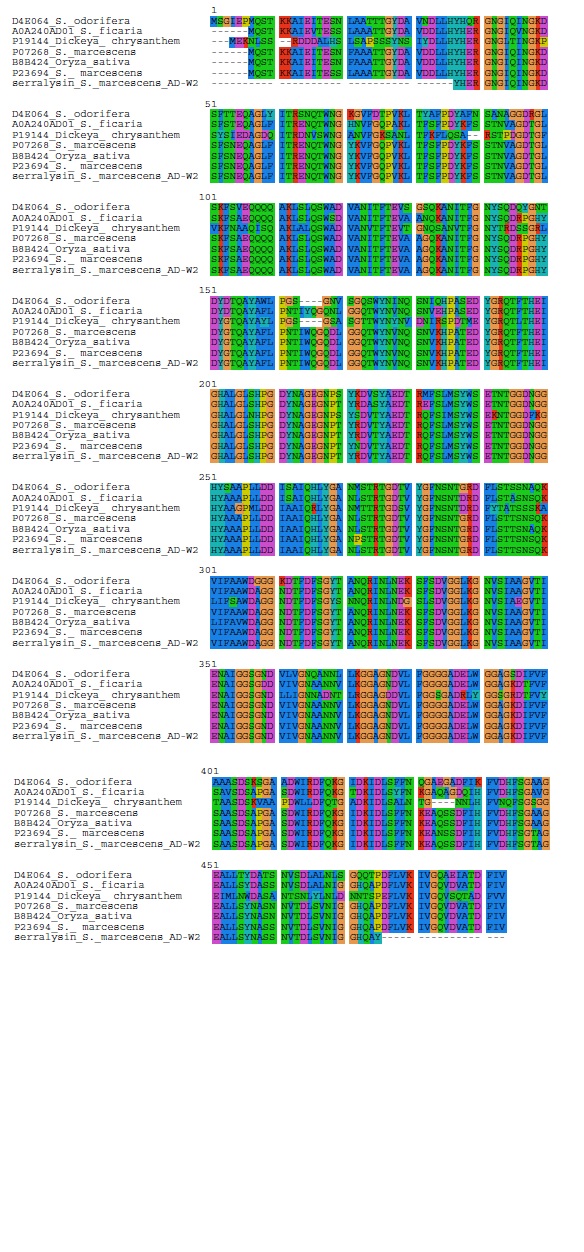


**Fig. S4** gene sequence of serralysin was translated into protein and then aligned with protein sequences having highest similarity in UNIPROT database, we could see that the Zinc binding domain is highly conserved in nature. Even in proteins predicted from the plant genome oryza sativa there is very high similarity with that from microbial sources. This might be an indication of close relation of Serratia marcescens in plants not only limited to mulberry (Koul et al. 2020) and Cucurbita pepa (Selvakumar et al. 2008) and also much greater role of serralysin as natural insecticidal in plants (Kaviyarasi and Suryanarayan 2016).

**References**

Kaviyarasi N, Suryanarayan V (2016) Serrapeptidase gene of *Serratia marcescens* from plant origin expressed by Pichia pastoris has protease activity. Indian J Med Res Pharm Sci 3:2349–5340

Laemmli UK (1970) Cleavage of structural proteins during the assembly of the head of bacteriophage T4. Nature 227:680–685

Koul D, Chander D, Manhas RS, Chaubey A (2020) Isolation and Characterization of Serratiopeptidase Producing Bacteria from Mulberry Phyllosphere. Curr Microbiol. https://doi.org/10.1007/s00284-020-02280-0

Perkins DN, Pappin DJ, Creasy DM, Cottrell JS (1999) Probability-based protein identification by searching sequence databases using mass spectrometry data. Electrophor Int J 20:3551–3567

Rausch T, Fritz MH-Y, Untergasser A, Benes V (2020) Tracy: basecalling, alignment, assembly and deconvolution of sanger chromatogram trace files. BMC Genomics 21:230 . https://doi.org/10.1186/s12864-020-6635-8

Selvakumar G, Mohan M, Kundu S, Gupta AD, Joshi P, Nazim S, Gupta HS (2008) Cold tolerance and plant growth promotion potential of *Serratia marcescens* strain SRM (MTCC 8708) isolated from flowers of summer squash (Cucurbita pepo). Lett Appl Microbiol 46:171–175 . https://doi.org/10.1111/j.1472-765X.2007.02282.x

Shevchenko A, Tomas H, Havli J, Olsen JV, Mann M (2006) In-gel digestion for mass spectrometric characterization of proteins and proteomes. Nat Protoc 1:2856–2860

Srivastava V, Mishra S, Chaudhuri TK (2019) Enhanced production of recombinant serratiopeptidase in *Escherichia coli* and its characterization as a potential biosimilar to native biotherapeutic counterpart. Microb Cell Factories 18:1–15
